# A Systematic Review of Risk factors for Sleep Apnea

**DOI:** 10.1016/j.pmedr.2024.102750

**Published:** 2024-05-03

**Authors:** Josef Yayan, Kurt Rasche

**Affiliations:** Department of Internal Medicine, Division of Pulmonary, Allergy, and Sleep Medicine, HELIOS Clinic Wuppertal, Witten/Herdecke University, Witten, Germany

**Keywords:** Sleep apnea, Risk factors, Obesity, Age, Gender, Neck circumference, Family history, Smoking, Alcohol use, Medical conditions, Nasal congestion

## Abstract

**Background:**

Sleep apnea, a prevalent global health issue, is characterized by repeated interruptions in breathing during sleep. This systematic review aggregates global data to outline a comprehensive analysis of its associated risk factors.

**Purpose:**

The systematic review underscores the global prevalence of sleep apnea and the universal importance of its early detection and management by delineating key risk factors contributing to its development.

**Material and Methods:**

We conducted a thorough systematic review of international medical databases up to July 31, 2023, including PubMed, Medline, and Cochrane Library, to ensure a wide-ranging collection of data reflective of various populations.

**Results:**

The systematic review identifies several risk factors such as obesity, age, gender, neck circumference, family history, smoking, alcohol use, underlying medical conditions, and nasal congestion, highlighting their prevalence across diverse demographics globally.

**Conclusion:**

Emphasizing lifestyle modifications and proactive interventions, our findings advocate for global health strategies to mitigate the risk of sleep apnea and enhance sleep health worldwide.

## 1 Introduction

Sleep apnea is a prevalent sleep disorder characterized by recurrent interruptions in breathing during sleep, thereby leading to fragmented sleep patterns and potential health risks (Eckert and Malhotra, 2008). It affects millions of individuals worldwide and has significant implications for their overall well-being and quality of life (Farhud, 2015). Understanding the risk factors associated with sleep apnea is crucial for early detection, prevention, and effective management of this condition (Young et al., 2002). This systematic review aims to provide an in-depth analysis of the key risk factors that contribute to sleep apnea, thereby highlighting their interplay as well as the implications of sleep apnea for public health. Sleep apnea is broadly classified into two main types: obstructive sleep apnea (OSA) and central sleep apnea (Malhotra and Owens, 2010). OSA, the most common form, occurs when the upper airway becomes partially or completely blocked during sleep (Anttalainen et al., 2016). On the other hand, central sleep apnea is result of a communication breakdown between the brain and respiratory muscles, thereby leading to interrupted breathing (Thalhofer and Dorow, 1997). Both types can have severe consequences, including daytime fatigue, impaired cognitive function, cardiovascular complications, and an increased risk of accidents (Jean-Louis et al., 2008, Cheng et al., 2021).

## 2 Material and methods

To compile relevant data for this systematic review, a comprehensive search of medical databases until 31st July 2023—including PubMed, Medline, and Cochrane Library—was conducted using keywords such as “sleep apnea,” “risk factors,” “obesity,” “age,” “gender,” “smoking,” “alcohol use,” and “medical conditions.” Studies addressing the association between sleep apnea and these risk factors were considered for inclusion in the present systematic review. Data from selected studies were extracted, analyzed, and synthesized to identify common risk factors and their impact on the development of sleep apnea.

### 2.1 Inclusion criteria

Only studies that explicitly discussed the association between sleep apnea and identified risk factors were included. Studies with clear data collection and analysis methods were considered.

### 2.2 Exclusion criteria

Studies not directly examining the relationship between sleep apnea and the specified risk factors were excluded. Studies with incomplete data or unclear methodologies were not considered. Unpublished studies or those not peer-reviewed were excluded to maintain the scientific validity of the systematic review.

### 2.3 Data extraction and synthesis

Selected studies were meticulously reviewed, and data pertinent to the scope of this research—such as participant demographics, study design, outcomes, and key findings—were extracted. This data was then analyzed and synthesized to identify prevalent risk factors and assess their influence on the development of sleep apnea. The compilation adheres to the PRISMA (Preferred Reporting Items for Systematic Reviews and Meta-Analyses) guidelines to ensure rigorous and standardized reporting.

## 3 Results

**Fig. 1:**
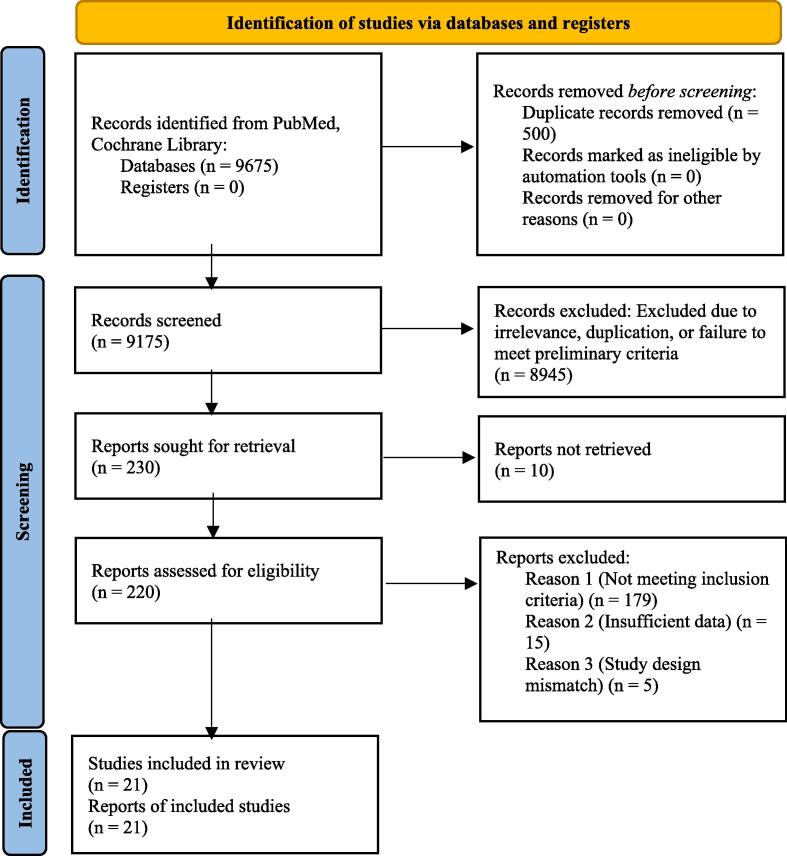
**Detailed PRISMA Flow Diagram of the Systematic Review Process for Identifying Key Studies on Sleep Apnea Risk Factors.** This flow diagram showcases the step-by-step methodology used in systematically identifying, screening, and evaluating studies to pinpoint those that effectively contribute to understanding the risk factors for sleep apnea, culminating in 21 high-quality studies included in the final systematic review.

Our systematic review, adhering to PRISMA standards, initially identified 9,675 potential studies. Through rigorous evaluation, we distilled this number to 21 key studies that best met our stringent criteria for relevance and quality (Fig. 1). The systematic review identified several prominent risk factors associated with sleep apnea (Table 1):

**Table 1:** **Publications about Risk Factors on Sleep Apnea.** This table summarizes key studies that investigate various risk factors associated with sleep apnea, focusing on the links between obesity, gender differences, genetics, and other demographic and physiological variables.

Author (Year)	Country	Study design	Total Number of participants	Outcome	Brief keywords
Jehan et al. (2017)	USA	Cross-sectional	89	Public health implications	Obesity, sleep apnea
Schwartz et al. (2008)	USA	Review	200	Therapeutic approaches	Obesity, pathogenesis
Ciavarella et al. (2018)	Italy	Retrospective	75	BMI correlation	BMI, sleep apnea
Alshehri et al. (2019)	Saudi Arabia	Cross-sectional	803	Severity pattern	Obesity, severity
Kripke et al. (1997)	USA	Survey	355	Prevalence	Age, prevalence
Punjabi (2008)	USA	Review	150	Epidemiology	Adults, epidemiology
Lin et al. (2008)	USA	Review	320	Gender differences	Gender, treatment
Kim et al. (2019)	South Korea	Cross-sectional	119	Gender onset differences	Gender, age differences
Huang et al. (2018)	USA	Prospective cohort	50,327	Menopause	Menopause, risk
Katz et al. (1990)	Canada	Cross-sectional	123	Neck circumference	Thick necks, risk
Ho et al. (2016)	USA	Cross-sectional	151	Neck circumference-height ratio	Neck ratio, prediction
Cielo et al. (2021)	USA	Prospective cohort	119	Neck fat	Neck fat, adolescents
Redline et al. (2000)	USA	Review	4028	Genetics	Sleep apnea, genetics
Szily et al. (2019)	Hungary	Twin study	300	Genetics	Genetics, sleepiness
Wang et al. (2015)	China	Cross-sectional	47	Inflammation	COPD, inflammation
Kaleelullah et al. (2021)	USA	Review	200	Lifestyle impact	Lifestyle, sleep apnea
Krishnan et al. (2014)	USA	Cross-sectional	350	Smoking relationship	Smoking, sleep apnea
Baik et al. (2015)	South Korea	Genome-wide association	1763	Genetic associations	Genetics, sleep apnea
Coussa-Koniski et al. (2020)	Lebanon	Retrospective	663	Epidemiology	OSA characteristics
Young et al. (1997)	USA	Population-based survey	4927	Nasal obstruction	Nasal obstruction, risk
Lu et al. (2005)	USA	Retrospective	151	Sedative effects	Sedatives, consequences

**1. Obesity:** Excess weight can lead to a narrowing of the airway, thereby increasing the risk of OSA (Jehan et al., 2017). Obesity emerged as a significant risk factor, with excess weight contributing to airway narrowing and the development of OSA (Schwartz et al., 2008). Studies consistently revealed a positive correlation between body mass index (BMI) and the severity of sleep apnea (Ciavarella et al., 2018, Alshehri et al., 2019).

**2. Age:** Sleep apnea becomes more prevalent with age, particularly in people over 40 (Kripke et al., 1997). Age was also found to be a critical factor in the studies analyzed, as the prevalence of sleep apnea increases with age due to natural changes in the upper airway and respiratory muscles (Punjabi, 2008).

**3. Gender:** Men are more likely to develop sleep apnea than women, although the risk for women increases if they are overweight or post-menopausal (Lin et al., 2008, Kim and Taranto-Montemurro, 2019). However, post-menopausal women with weight gain demonstrated an increased susceptibility, thereby suggesting that hormonal changes may play a role in the development of sleep apnea (Huang et al., 2018).

**4. Neck circumference:** A thick neck may possible narrow the airways, increasing the likehood of developing sleep apnea (Katz et al., 1990). Neck circumference has been identified as an independent risk factor for sleep apnea (Ho et al., 2016). Individuals with a thicker neck may experience increased airway complexity, leading to higher susceptibility to be condition (Cielo et al., 2021).

**5. Family history:** The occurrence of sleep apnea in family members may be a risk factor (Redline and Tishler, 2000). Family history emerged as a potential genetic predisposition in the studies analyzed, thereby indicating that a family history of sleep apnea may increase an individual’s likelihood of developing the disorder (Szily et al., 2019).

**6. Smoking and alcohol use:** Both smoking and alcohol consumption can contribute to airway inflammation and muscle relaxation, thereby worsening sleep apnea symptoms (Wang et al., 2015). The analyzed studies revealed that lifestyle factors were found as a risk factor for sleep apnea (Wang et al., 2015, Kaleelullah and Nagarajan, 2021). Smoking and alcohol worsened sleep apnea symptoms (Krishnan et al., 2014, Baik et al., 2015).

**7. Medical conditions:** Chronic disorders such as hypertension, diabetes, and hearth failure are risk factor for sleep apnea (Coussa-Koniski et al., 2020).

**8. Nasal congestion:** Chronic nasal congestion can obstruct airflow during sleep (Young et al., 1997). Nasal polyps, and chronic sinusitis lead to breathing difficulties during sleep (Young et al., 1997).

**9. Use of sedatives or tranquilizers**: These medications can lead to breathing difficulties during sleep through relax throat muscles (Lu et al., 2005).

## 4 Discussion

The results of this systematic review showed the multifactorial cause for the development of sleep apnea. More risk factors together can increase the likelihood of sleep apnea developing.

Obesity is a changeable risk factor for sleep apnea. Weight control measures are of great importance in the prevention of sleep apnea (Tham et al., 2019). Obesity is a main cause for sleep apnea, particularly for OSA (Jehan et al., 2017). Fat accumulation around the neck can narrow the upper airway and interfere with ventilation during sleep (Schwartz et al., 2008). Additionally, obesity leads to inflammation, changes in hormones, and respiratory muscle dysfunction (Schwartz et al., 2008). Effective weight management strategies—including lifestyle changes and behavioral interventions—have shown promise in improving sleep apnea outcomes, thereby emphasizing the importance of addressing obesity in the management of sleep apnea (Tham et al., 2019).

Sleep apnea can be improved with weight control, lifestyle changes, and behavioral interventions (Tham et al., 2019). In addition, sleep apnea is favored by aging processes in the airways and respiratory muscles in older people (Oliven et al., 2001). Moreover, understanding the gender-specific risks and hormonal influences can guide tailored approaches for risk assessment and management (Martins and Conde, 2021). The relationship between age and sleep apnea is complex (Addison-Brown et al., 2014). As individuals age, structural and functional changes occur in the upper airway and respiratory muscles (Oliven et al., 2001). These changes can lead to airway collapse, contributing to the development or worsening of sleep apnea (Pham and Schwartz, 2015). The comorbidities increase the risk of developing sleep apnea in elderly (Silva et al., 2022). Identifying risk factors for sleep apnea is of particular importance for the diagnosis of sleep apnea in the elderly (Quinnell and Smith, 2004).

Gender differences in the prevalence of sleep apnea are well-known (Lin et al., 2008). Men suffer from sleep apnea more often than women. This difference is partially attributed to anatomical variations, as men typically have narrower upper airways than women (Lin et al., 2008). Estrogens have a protective effect against sleep apnea (Galvan et al., 2017). The risk of sleep apnea after menopause increases (Huang et al., 2018).

Further, a thicker neck is an increased risk of sleep apnea (Ho et al., 2016). The accumulation of fat around the neck can lead to a more collapsible airway during sleep, making individuals more susceptible to OSA (Schwartz et al., 2010). Neck circumference measurement is simple for assessing the risk of sleep apnea, particularly in individuals who may not present with other obvious risk factors (Ho et al., 2016).

Sleep apnea could be a genetic condition. There is the need for further genetic studies to elucidate the underlying mechanisms and identify potential susceptibility genes (Mukherjee et al., 2018). While specific genetic factors contributing to sleep apnea are still being investigated (Mukherjee et al., 2018). Confirming sleep apnea as a genetic sleep disorder could help early detection in at-risk individuals (Redline and Tishler, 2000).

Lifestyle modifications, such as smoking cessation and alcohol moderation, are essential components of sleep apnea management (Duan et al., 2022). Additionally, addressing medical conditions and nasal congestion may lead to improved sleep apnea outcomes (Paidi et al., 2022). Smoking and alcohol could be avoided (Wetter et al., 1994, Simou et al., 2018). This would reduce the risk of sleep apnea. Smoking causes airway muscle inflammation. The inflammation of muscle leads to narrowing of the airways (Ioannidou et al., 2021). Alcohol increases the likelihood of airway collapse through muscle relaxation (Simou et al., 2018). Stopping smoking and avoiding alcohol are essential measures in the treatment of sleep apnea (Kaleelullah and Nagarajan, 2021).

In addition, some chronic disorders are an increased risk for sleep apnea. Heart failure, stroke, diabetes, and hypertension are the disorders usually associated with sleep apnea (Jean-Louis et al., 2008). These conditions can exacerbate structural and physiological changes in the airway respiratory system, thereby leading to sleep-disordered breathing (Jean-Louis et al., 2009). Recognizing and managing these medical comorbidities are essential components of comprehensive sleep apnea care (Jean-Louis et al., 2009). Moreover, chronic sinusitis, and nasal polyps are main the risk factors for sleep apnea (Jiang et al., 2016). Addressing nasal congestion through appropriate medical interventions may improve nasal airflow and subsequently alleviate sleep apnea (Michels Dde et al., 2014).

## 5 Limitations

This systematic review article provided a valuable overview of risk factors for sleep apnea. However, the results of the systematic review have some limitations that cannot be neglected. There may be a selection bias across the studies selected. Some studies may have had small sample sizes. The results of the systematic review cannot be generalized. The result of the interaction of various risk factors on sleep apnea requires further studies.

## 6 Conclusion

In conclusion, this systematic review provides a deeper understanding of the risk factors that contribute to sleep apnea. Sleep apnea is a multifaceted disorder with diverse risk factors interacting to contribute to its development and severity. A comprehensive understanding of these risk factors is vital for health care professionals to accurately assess individual risk profiles. By identifying and addressing these factors, health care professionals can improve sleep apnea management and overall sleep health of those suffering from sleep apnea. Early intervention, lifestyle modifications, and targeted approaches are crucial in reducing the burden of sleep apnea and its associated health complications. Tailored interventions that target modifiable risk factors—such as weight management, smoking cessation, and alcohol moderation—hold promise in reducing the burden of sleep apnea. Further research is warranted to unravel the intricate interplay of risk factors and develop effective prevention and treatment strategies for this prevalent sleep disorder. Additionally, further research into genetic predispositions and hormonal influences may pave the way for more effective and personalized approaches to sleep apnea prevention and treatment. Ultimately, early recognition and management of sleep apnea risk factors can lead to improved patient outcomes and overall sleep health.

## Funding

This research did not receive any specific grant from funding agencies in the public, commercial, or not-for-profit sectors.

## CRediT authorship contribution statement

**Josef Yayan:** Writing – review & editing, Writing – original draft, Visualization, Validation, Supervision, Software, Resources, Project administration, Methodology, Investigation, Funding acquisition, Formal analysis, Data curation, Conceptualization. **Kurt Rasche:** Writing – review & editing.

## Declaration of competing interest

The authors declare that they have no known competing financial interests or personal relationships that could have appeared to influence the work reported in this paper.

## Data Availability

The data that has been used is confidential.
